# Unveiling the grip of mobile phone addiction: an in-depth review

**DOI:** 10.3389/fpsyt.2024.1429941

**Published:** 2024-10-02

**Authors:** Jinyu Li, Hong Yang

**Affiliations:** School of Medical and Life Sciences, Chengdu University of Traditional Chinese Medicine, Chengdu, China

**Keywords:** mobile phone addiction, assessment, mechanism, treatment, pathophysiology

## Abstract

Mobile Phone Addiction represents an emergent addictive disorder that gravely jeopardizes the physical and mental health of adolescents worldwide, necessitating exhaustive research. Current reviews of MPA are in dire need of updates and enhancements. Therefore, this review aggregates the extant research spanning the past two decades on the prevalence, pathogenesis, comorbidities, assessment, and treatment of MPA, aiming to furnish a reference for future investigations into this condition.

## Graphical Abstract

Pathogenesis, pathophysiology, assessment and treatment of MPA.

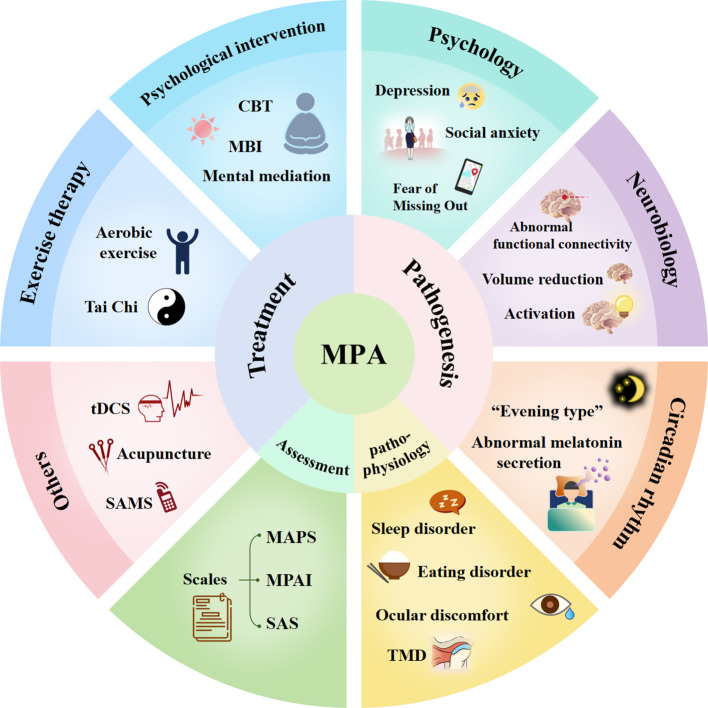

## 1 Introduction

Mobile phone is becoming a necessary tool for daily life, changing living, working, and leisure activities (1). It is a double-edged sword one side offers convenience, but the other side could seriously damage physical and mental health when overused (2). Mobile Phone Addiction (MPA) refers to excessive use of mobile phones by individuals, which leads to a loss of self-control over mobile phone use, affecting social function and causing psychological or behavioral problems, and is characterized by withdrawal, tolerance, and loss of control (3). MPA is alternatively denominated as Mobile Phone Overuse (MPO) (4), Problematic Smartphone Use (PSU) (5), and Mobile Phone Addiction Tendency (MPAT) (6). Recent data from June 2021 underscores a significant adoption of mobile internet usage among individuals aged 10 to 19 in China, with the user base reaching a staggering 123.86 million (7). Compared to other age groups, adolescents exhibit a heightened risk for MPA (8).

Current research on the mechanisms of MPA can be broadly categorized into three main areas. The first is psychological mechanisms. This area primarily focuses on psychological factors, including anxiety, depression, and other mental health conditions. Research in this category predominantly employs qualitative methods to investigate the causal relationship between MPA and psychological disorders (9). Second, neurobiological mechanisms. The neurobiological underpinnings of MPA, include the role of the brain’s dopamine system and reward system. The application of neuroimaging techniques, such as functional magnetic resonance imaging (fMRI), has shed light on the neural mechanisms underlying MPA (10). The last one is emerging research with new technologies. This area encompasses studies using novel techniques and methods, such as genomics and metabolomics, to identify biological markers of MPA. These studies have yielded promising results, suggesting potential genetic susceptibility or aberrant gene methylation in MPA (11, 12). Additional insights: Research on associated conditions of MPA has revealed that sleep disorders are common among individuals with MPA. Moreover, analyses of saliva metabolites and microbiome composition in MPA individuals suggest potential involvement in the development of the disorder (13, 14). However, a significant gap exists in connecting neurobiological mechanisms with genetic susceptibility, microbiome, and other potential factors. Future research could explore the role of exosomes as a potential intermediary link in these complex interactions.

Presently, a globally unified diagnostic criterion for MPA is absent. Analogous to other behavioral addictions, assessment predominantly relies on evaluative scales, including but not limited to the Mobile Phone Addiction Scale (MAPS) (15), Mobile Phone Addiction Index (MPAI) (16), Smartphone Addiction Scale (SAS) (17), and others of a similar nature. Research into the treatment of MPA predominantly focuses on psychological (18), exercise (19), and various other interventions (20), yet these modalities exhibit significant limitations regarding the scope of treatment options and the durability of their efficacy. In this review, we aim to elucidate the recent founding of MPA mechanism, pathophysiology, assessment, and intervention, and discuss the current limitations.

## 2 Methods

This review employed an in-depth approach to comprehensively examine the multifaceted phenomenon of MPA. We aimed to provide a nuanced understanding of the topic, considering both empirical findings and theoretical perspectives. The following methods were employed. Search strategy: A systematic search of the literature was conducted using the following electronic databases: PubMed, PsycINFO, Embase, and Web of Science. The search terms included combinations of keywords related to “mobile phone addiction”, “smartphone addiction”, “problematic mobile phone use”, “neuroimaging”, “brain structure”, “brain function”, “behavioral correlates”, “Treatment”, “Therapy”, “Therapeutic approach”, “Intervention Pathogenesis”, and “Mechanism”. The search was not limited by publication date, but only articles published in English were considered. In addition to the database searches, the reference lists of relevant review articles and the authors’ files were manually searched to identify any additional eligible studies. Gray literature, such as conference proceedings and dissertations, was also screened to minimize publication bias. The following inclusion criteria were applied: (1) Studies focused on mobile phone addiction, its causes, consequences, or prevention and treatment strategies. (2) Studies involving human participants of any age, from diverse cultural and geographical backgrounds. (3) English-language studies, including both peer-reviewed and non-peer-reviewed sources. The following exclusion criteria were applied: (1) Studies unrelated to mobile phone addiction or lacking clear connections to the topic. The synthesis was organized into thematic sections, including the neurobiological correlates of mobile phone addiction, the psychological and behavioral features, and the diagnostic and assessment instruments used in this field. Limitations of the current evidence base and directions for future research were also discussed. To capture the breadth of the topic, we searched for studies published from 2010 onwards.

## 3 Risk factors for MPA

The prevailing consensus within the scholarly community suggests that psychological and neurobiological factors are central to the disorder’s etiology. Emerging evidence indicates that genetic basis and circadian rhythm may also play a role in MPA (21).

### 3.1 Mental health issues

Compensatory Internet Use Theory (CIUT) posited that depression and anxiety may intensify mobile phone usage resulting in MPA (22). In recent years, research has focused on the psychology and MPA (23, 24). Especially anxiety and depression served as significant predictors of MPA (25).

#### 3.1.1 Depression

Depression is characterized by an enduring state of low mood and a significant reduction in self-esteem (26). Employing network analysis, Wei (27) elucidated the linkage between symptoms of MPA and depression. Their findings were congruent with the CIUT tenets. They contended that MPA represented not merely a tactic to elude depressive moods but also a consequential outcome of depression, which incited a loss of interest in alternative activities among individuals. Nevertheless, some research posited that MPA positively predicted the manifestation of depressive symptoms in individuals at a later stage, yet initial depressive symptoms appeared to bear no relevance to subsequent MPA occurrences (28). Furthermore, a cyclical interplay may be present between MPA and depressive symptoms (29). Engagement with mobile phones may have transiently alleviated negative emotional states, including depression, thereby reinforcing usage patterns. However, when access to mobile phones was impeded, individuals experienced negative emotions due to unfulfilled cravings. The reward system in the neural network was less active in MPA patients, a phenomenon paralleled in patients with depressive disorders (30). Nevertheless, a regression analysis study indicated that the link between MPA and depression was not mediated by the reward system. Depression and reward processing emerged as unique predictors of MPA, they did not share a common mechanism (31).

#### 3.1.2 Anxiety

Social anxiety refers to the experience of discomfort, fear, and agitation in social situations, particularly in settings requiring face-to-face interaction (30). In an attempt to alleviate anxiety, those particular individuals exhibiting social anxiety depicted a preference for interpersonal communication via mobile phones, subsequently rendering them more susceptible to MPA (32). The research conducted by Li (33) indicated that shyness acted as a predictive factor for MPA, given that individuals with shyness were more susceptible to the onset of social anxiety disorder. Shyness was considered a type of social disengagement (34). Three varieties of social withdrawal were identified by social motivation theory: social evasion, shyness, and social disorder (35). In contrast to individuals who had social evasion and social disorder, individuals with shyness exhibited a paradoxical profile with higher motivations for social engagement and higher inclinations for social withdrawal. Therefore, within these subtypes, shyness was the most distinctly contradictory. This internal conflict led to social anxiety, prompting shy individuals to prefer mobile phone-mediated communication over face-to-face interactions (32). Similarly, research has indicated that alexithymia (36) and low self-esteem (33) were likewise pivotal factors contributing to MPA. Individuals with alexithymia are unable to identify or describe their own emotions, and individuals with low self-esteem exhibit an exaggerated sensitivity to negative appraisals from others. Both groups, facing challenges in communicating with others, demonstrated heightened social anxiety. Consequently, they showed a preference for engaging in virtual social interactions via mobile phones to fulfill their social needs, thereby alleviating their anxiety. All these findings are congruent with the CIUT (22), which posited that mobile phone usage served to meet psychological needs and mitigate negative emotions.

The link between MPA and social anxiety and depression likely involves several mechanisms. One key factor is constant phone use replaces real-life interactions. This is further fueled by Fear of Missing Out (FOMO), the anxiety of missing out on social experiences, leading to a constant need to stay connected (37, 38). Tao (39) found that FOMO mediates the relationship between social anxiety and MPA. FOMO is a cognitive bias related to phone use, influencing emotional and mental responses. Executive functions and inhibitory control then influence decision-making regarding phone use. In essence, social anxiety can lead to FOMO, which in turn drives excessive phone use and contributes to MPA, potentially exacerbating social anxiety and depression (40).

### 3.2 Neurobiological issues

#### 3.2.1 Brain connectivity in MPA

Dopamine (DA) and serotonin (5-HT) are considered crucial neurotransmitters within the brain’s reward system (41). The reward system consists of three key pathways: the mesolimbic dopamine pathway, the mesostriatal pathway, and the mesocortical pathway. Neuroimaging techniques, such as fMRI, have revealed the neurobiological basis of MPA. Research using neuroimaging techniques has identified several brain regions associated with MPA: 238 Chinese university students’ brains MRI indicated that MPA exhibited an augmented intrinsic functional connectivity (IFC) between several cortical regions (28). Li (42) used functional near-infrared spectroscopy (fNIRS) to compare brain activity in individuals with MPA and healthy controls. They found reduced brain activity and functional connectivity in the creative idea-generation process in individuals with MPA. Pyeon demonstrated reduced connectivity between the right inferior frontal gyrus and limbic regions in individuals with MPA (43). This reduced connectivity was associated with MPD severity, self-control levels, and the amount of time spent using mobile phones. Horvath observed a significant negative correlation between MPD and the volume and activity of the right anterior cingulate cortex (44). Han found that activation levels in the frontal pole cortex (FPC) were significantly negatively correlated with MPA (45). The FPC plays a crucial role in the self-regulation of attention, suggesting a neurobiological mechanism for the difficulty in focusing attention experienced by individuals with MPA. Studies have identified (46) that there was a notable reduction in functional connectivity (FC)within the salience and central executive networks among individuals with MPA. This diminution was characterized by reduced FC among the front insular cortex, dorsolateral prefrontal cortex (DLPFC) and the ventrolateral prefrontal cortex (VLPFC). These studies collectively highlight the involvement of specific brain regions and pathways connection in the development and maintenance of MPA.

#### 3.2.2 Brain substructure and volumes in MPA

**Figure 1:**
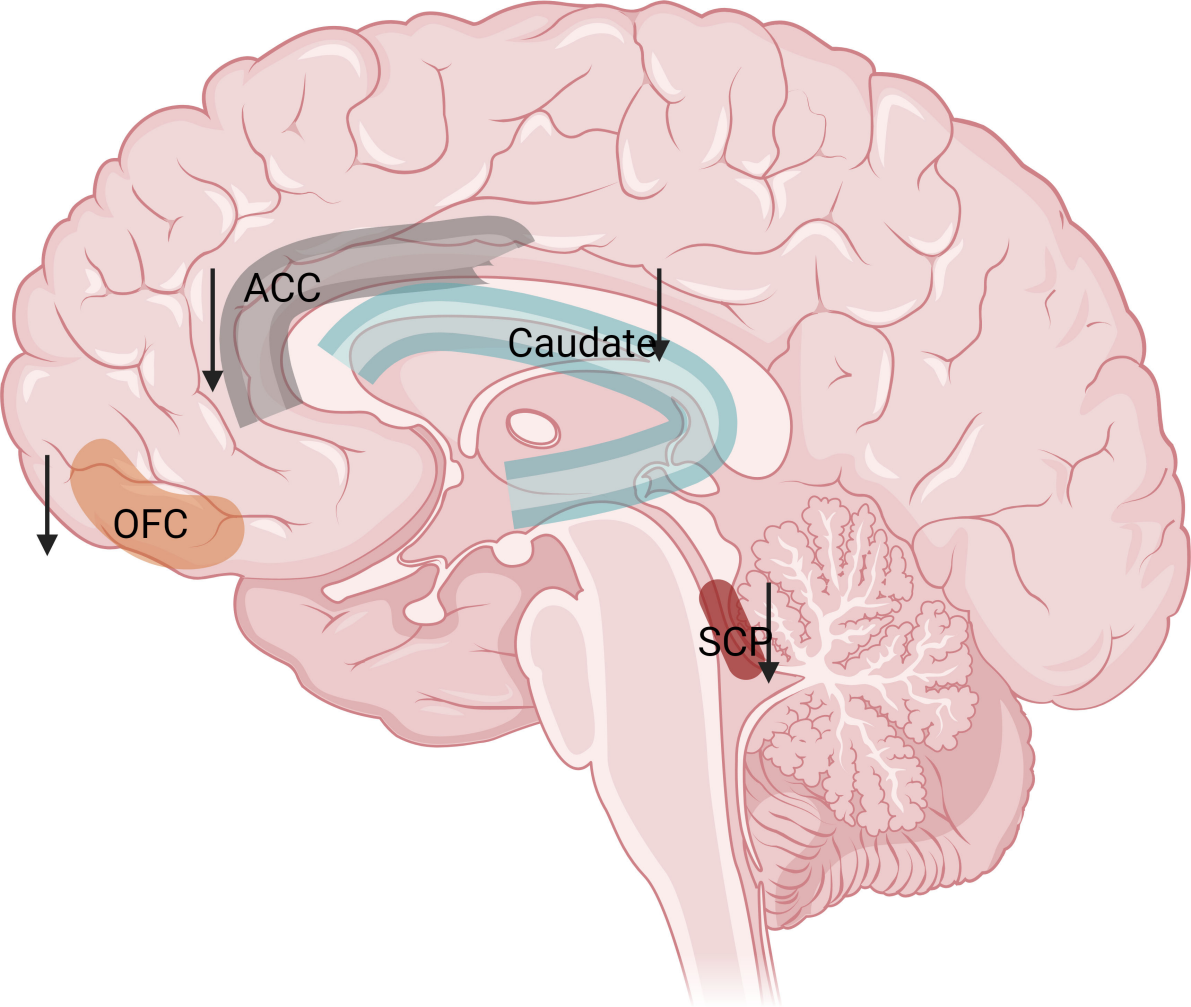
Volume reduction of the caudate nucleus (related to compulsivity)、ACC (decrease error monitoring and cognitive control abilities)、SCP (Interfering with the balanced regulation of reward and aversion)、OFC (disrupt adaptive decision-making processes) in MPA. The figure was created by Biorender.

Research on the brains of people with MPA has revealed changes in several key regions: Caudate Nucleus, an area associated with reward and impulsivity, was smaller in the MPA group than in the normal group, and the degree of shrinkage was positively correlated with the severity of MPA (47). Lateral Orbitofrontal Cortex (OFC), this area is crucial for decision-making and impulse control (48). People with MPA have less gray matter in this region, which may contribute to their difficulty in making choices (49). Anterior cingulate cortex (ACC), this region is involved in reward processing and error monitoring. Studies suggest that it may be less active in individuals with MPA, potentially impairing their ability to adjust their behavior. Superior Cerebellar Peduncle (SCP), this brainstem structure is involved in transmitting signals related to motivation. People with MPA have smaller SCP volumes, which may be linked to difficulties with reward and aversion (50). These findings implicate the reduction in SCP volume as a contributory neurobiological mechanism underlying MPA. These brain changes may play a role in the development and maintenance of MPA. The volume reduction in MPA is shown in **Figure 1**.

#### 3.2.3 Brain Activation in MPA

Chun’s research showed (51) that when MPA patients were faced with other people’s negative emotions and emotional changes, the dorsolateral prefrontal cortex (DLPFC) and dorsal anterior cingulate cortex (DACC) activation was reduced. The DLPFC and DACCA are involved in cognitive control and emotion regulation (52). Their reduced activation indicated that individuals had cognitive defects in processing other people’s facial emotions during social interactions, which in turn led to poor real-life social experiences and prompted individuals to meet their social needs through virtual social interactions on mobile phones.

### 3.3 Genetic and circadian rhythm issues

Circadian rhythm is a biological rhythm with an approximately 24-hour cycle that enables organisms to adapt to the daily fluctuations inherent in the day-night cycle, thereby maintaining an internal temporal alignment with external environmental changes (53). Individuals’ circadian preferences constituted a significant predictive factor for MPA (21) and were categorized into three distinct phenotypes (54): the “morning type,” “evening type” and “neither type.” Individuals identified as the evening type were predisposed to spending more on social interactions (55) and were at a higher risk of developing MPA, aligning with the notion posited by Walsh that mobile phones served as a social facilitator (56). Additionally, evening-type individuals were more inclined to delay their sleep, resorting to mobile phone usage to alleviate the stress induced by postponed sleep, thereby seeking immediate gratification (57). Moreover, using mobile phones at night increased individuals’ exposure to light, intensified their propensity towards the evening and further augmented the likelihood of developing MPA (58). Furthermore, melatonin not only played a pivotal role in modulating circadian rhythms but also served as a “state marker” and “trait marker” for emotional regulation (59). Disruptions in circadian rhythms could precipitate aberrant secretion of melatonin, leading to affective dysregulation (such as depression) (60), which may subsequently have contributed to the development of MPA.

## 4 Pathophysiology of MPA

### 4.1 Sleep disorder

Sleep disorders (SD) encompass difficulties in initiating sleep, insufficient sleep duration, insomnia, and sleeplessness (61). MPA has become a pivotal contributor to the onset of sleep disturbances (62). Cross-sectional research elucidated that MPA indirectly compromised sleep quality by inducing pre-sleep delay (63). This finding concurs with Steel’s Temporal Motivation Theory (TMT), wherein an individual’s tendency to procrastinate is contingent on the perceived utility of a task (62). MPA, the time required to reap the health benefits of sleep is considerably more significant than the time needed to attain immediate gratification from using a mobile phone. Consequently, individuals with MPA were predisposed to defer the task of sleeping in favor of a relatively heightened perceived utility. Additionally, research has revealed that utilizing a mobile phone during intended sleep hours can alter circadian rhythm systems, cerebral blood flow, and heart rate, thereby negatively impacting sleep quality (64).

### 4.2 Eating disorder

An eating disorder constitutes a pathological condition characterized by aberrant eating behaviors or weight control practices, which pose a severe threat to physical well-being (65). Cross-sectional research (66) has illuminated a positive correlation between MPA and eating disorders, though the research could not ascertain a causal linkage between the two phenomena. A subsequent longitudinal investigation, spanning one year and encompassing 1,181 university students, elucidated that MPA precipitated eating disorders rather than the control (67). This could potentially be correlated with the increased susceptibility of individuals with MPA to sociocultural influences. The pervasive dissemination of slimming and beauty marketing through digital platforms frequently engendered dissatisfaction among individuals with their physiques and appearances (68). The portrayal and propagation of an idealized body on social media further amplifies body anxieties. Consequently, adolescents who regularly engaged in selfie-taking and shared these photos on social media platforms were more prone to manifest symptoms of eating disorders (69). Another contributory factor is that MPA alters lifestyle-related elements, culminating in irregular eating patterns. Research identified a significant positive correlation between MPA and the frequency of consuming late-night snacks, fast food, and carbonated soft drinks (70). Furthermore, radio frequency-modulated electromagnetic fields (RF-EMFs) emitted by mobile phones have been linked to a notable increase in food intake, particularly that of carbohydrates (71), representing a pivotal element in the etiology of eating disorders. Synthesizing these factors, it can be hypothesized that individuals with MPA, being chronically exposed to RF-EMFs, may develop deleterious dietary habits such as late-night snacking and frequent fast food consumption, consequently leading to eating disorders in this demographic.

### 4.3 Somatic symptom

MPA is frequently associated with reduced levels of physical activity. A study targeting Chinese international students in South Korea revealed (72) that the severity of MPA was inversely correlated with the average daily step count. The study reveals that individuals addicted to mobile phones exhibit a significant reduction in daily walking steps, coupled with an increase in body fat and a decrease in muscle mass. This indicates that MPA negatively impacts health by reducing physical activity levels, such as walking. Additionally, the research finds a positive correlation between the duration of daily mobile phone use and the degree of MPA, while there is an inverse relationship between the number of daily walking steps and the level of MPA. Research indicated that prolonged use of mobile phones was associated with the occurrence of neck pain (73), and individuals with chronic neck pain demonstrated a higher prevalence of cervical intervertebral disc degeneration (74). This condition was correlated with maintaining a flexed head posture for extended periods during mobile phone use (75). A study found that individuals with MPA experienced more severe and frequent symptoms of pain-related temporomandibular disorders (TMD) (76), which may also have been related to the long-term maintenance of a head-forward posture by individuals with MPA. Additionally, individuals afflicted with MPA tended to engage with their mobile phone screens for more extended periods than the average person (77). Consequently, this cohort exhibited a greater prevalence of ocular discomfort, including symptoms such as redness, dryness, and visual disturbances. The incidence rate of these ocular symptoms was correspondingly higher in individuals with MPA than in the general population.

## 5 Assessment of MPA

MPA has not been included in the Diagnostic and Statistical Manual of Mental Disorders (78). The assessment for MPA primarily relies on assessment scales developed by various researchers tailored to the characteristics of distinct populations (refer to **Table 1** for details). Among these, the Mobile Phone Addiction Scale (MAPS) (15), the Mobile Phone Addiction Index (MPAI) (16), and the Smartphone Addiction Scale (SAS) (17) are extensively utilized in research on MPA.

**Table 1:** MPA-related scale.

Scale	Time	Items	Sample	Factors	Reliability (α)
Mobile Phone Addiction Scale (MPAS) (17)	2012	11	Female college students	3	0.86
Mobile Phone Addiction Index (MPAI) (18)	2008	17	Adults and teenagers	4	0.90
Smartphone Addiction Scale (SAS) (19)	2013	33	Adults (ages 18 to 53)	6	0.97
Smartphone Addiction Scale- Short Version(SAS-AV) (67)	2013	10	Adolescents	1	0.91
The Smartphone Addiction Proneness Scale (SAPS) (82)	2014	15	Adolescents	4	0.88
Mobile Phone Problem Use Scale (MPPUS) (66)	2005	27	Adults	/	0.93
Mobile Phone Dependence Questionnaire (MPDQ) (83)	2006	20	College students	1	0.86
Cellular Phone Dependence Questionnaire (CPDQ) (5)	2004	20	College students	6	0.86
Mobile Phone Usage Scale (MPUS) (84)	2007	33	College students	6	Factor analysis
Cell-Phone Addiction Scale for Korean Adolescents (CPAS) (85)	2009	20	Adolescents	3	0.92
Excessive Cellular Phone Use Survey (ECPUS) (86)	2008	20	Adolescents	/	0.87
SMS Problem Use Diagnostic Questionnaire (SMS-PUDQ) (87)	2007	8	Primary school student	2	0.84–0.87
Problem Cellular Phone Use Questionnaire (PCPU-Q) (88)	2009	12	Adolescents	/	0.85
Mobile Phone Involvement Questionnaire (MPIQ) (89)	2010	8	Teenagers and younger adults	1	0.78
Cell-Phone Addiction Assessment Questionnaire (KBUTK) (90)	2011	33	Students in elementary school and college	4	0.91
Problematic Use of Mobile Phones (PUMP) Scale (91)	2013	20	Adults (age 18 to 75)	1	0.94
Mobile Phone Activities and Addiction of Parents (MPAA) (92)	2014	21	Guardians of pupils	7	0.91
Smartphone Addiction Inventory (SPAI) (68)	2014	26	College students	4	0.94

### 5.1 Mobile Phone Addiction Scale

Hong (13) fashioned MPAS predicated on Young’s (79) Internet Addiction Test, devised to assess the severity of MPA. The scale encompassed 11 items across three dimensions: time management issues, academic difficulties and their impacts, and substitution of reality. It employed a six-point scoring system, with 1 denoting “strongly disagree” and 6 indicating “strongly agree.” Higher aggregate or mean scores suggested a more severe degree of MPA. Furthermore, according to Young’s Internet Addiction Test diagnostic criteria, item scores between 4 to 6 denoted prominent symptoms, and pronounced symptoms in seven or more items may have warranted a diagnosis of MPA.

### 5.2 Mobile Phone Addiction Index

Building upon the foundations laid by Bianchi and Phillips’ Problematic Mobile Phone Use Scale (80), Leung (16) constructed MPAI to diagnose symptoms of MPA. The Likert scoring system used by this scale has five points: 1 for “strongly disagree” and 5 for “strongly agree.” The 17 items were divided into four categories: compulsivity, withdrawal, escape, and inefficiency. A higher total or average score correlated with a more acute degree of MPA. Drawing on Young’s criteria for screening internet addiction, affirmative responses to 8 specific items were sufficient to categorize an individual as an MPA sufferer (81).

### 5.3 Smartphone Addiction Scale

Kwon (17) developed the first Smartphone Addiction Scale (SAS), which considered the distinctive attributes of mobile phones and was aimed at evaluating mobile phone usage to determine proclivities toward mobile phone addiction without rendering a clinical diagnosis. The scale comprised 33 items distributed across six dimensions: withdrawal, tolerance, positive anticipation, overuse, disruption of daily life, and cyberspace-oriented relationships. It utilized a six-point rating system, 1 signified “strongly disagree,” whereas 6 conveyed “strongly agree.” Higher total or average scores indicated an increased degree of MPA. **Table 1** shows the MPA-related scale.

## 6 Treatment of MPA

From the mechanism of MPA, it is understood that MPA results from a combination of external and internal factors. Consequently, current treatments for MPA encompass psychological intervention, exercise therapy, and technical intervention, as shown in **Table 2**.

**Table 2:** Interventions for MPA.

Category	Intervention means	Intervention object	Intervention results
Psychological intervention	CBT (94)	A total of 95 MPA college students were divided into three groups at random: the control group (34 individuals), the mind-body exercise (ME) group (31 individuals), and the CBT group (30 individuals)	ME and CBT effectively reduced the level of MPA
	indigenously adapted cognitive–behavioral therapy (IACBT) (69)	Students (12–19 years old) were divided into two groups: the IACBT group (62 participants) and the brief educational group (62 participants)	The IACBT group showed a significant reduction in MPA along with a decrease in symptoms of anxiety, stress, sadness, hyperactivity, and emotional issues
	CBT (95)	42 Chinese college students were split into two groups at random: the wait-list control condition and the group CBT intervention	The outcome revealed that compared to the wait-list controls, the CBT group showed a substantially more significant decrease in MPA
	Brief mindfulness training (96)	A control group and a mindfulness training group consisting of 22 college students each	College students’ MPA was effectively reduced and their feeling of purpose in life increased with brief mindfulness training
	Mindfulness-Based Mental Health Education Therapy (97)	Based on the evaluation results, 56 adolescent participants were chosen, and 28 people each were placed in the experimental and control groups at random	Teenagers with MPA benefited greatly from mindfulness-based mental health education therapy, which also enhanced their mental and cognitive faculties
	group mindfulness-based cognitive-behavioral intervention (GMCI) (71)	A control group of 29 university students with MPA and an intervention group of 41 participants were formed	University students’ MPA considerably reduced by GMCI
	Mindfulness-based cognitive programme(MBCP) (98)	A total of 240 elementary school pupils were divided equally between the intervention and control groups at random.	MBCP led to improvements in resilience and reduction of MPA risk among adolescents
	Mind subtraction meditation (72)	Experimental group (24 individuals) and control group (25 individuals)	Mind subtraction meditation on MPA in adolescents has a positive effect.
	Mind subtraction meditation (99)	experimental group (24 primary school students) and control group (22 primary school students),	Mind subtraction meditation improved mental health and decreased MPA
Exercise intervention	Aerobic exercise and Tai Chi Chuan (75)	the aerobic exercise group (30 individuals) the Tai Chi Chuan group (30 individuals) the wait-list control group (30 individuals)	AE and TCC reduced MPA
	Moderate-intensity aerobic exercise (77)	the control group (30 individuals) and the exercise group (30 individuals), with equal representation by major and gender	Moderate-intensity aerobic exercise served as a dependable and successful treatment option for MPA.
	group-based basketball and Baduanjin exercise (100)	96 Chinese college students with MPA were randomly assigned to an excise group and a control group	Significant reductions in MPA were shown by both exercise treatments.
Technological intervention	tDCS (101)	College students Addiction group(39 people) Control group(41people)	tDCS significantly enhanced inhibitory control function in the addiction group
	SAMA (81)	14 adults aged 19-50	/
	Acupuncture (102)	Six patients in MPA with sleep disorder (MPASD) and six healthy controls	Restored quantity and rhythmicity of representative salivary metabolites in MPASD

### 6.1 Psychological intervention

In the array of psychological intervention strategies, Cognitive-behavioral therapy (CBT), Mindfulness-based intervention (MBI), and mental meditation emerge as principal approaches. CBT is a short-term, structured psychotherapeutic treatment designed to alleviate adverse emotions and addictive behaviors by modifying an individual’s erroneous cognitions and behavioral patterns (18). A 12-week randomized controlled trial ascertained that CBT significantly mitigated stress among university students, thereby alleviating the severity of MPA (93). Findings from a single-blinded randomized trial indicated that a three-month CBT intervention substantively alleviated MPA and enhanced psychological health (94). MBI, representing mindfulness, fosters psychological well-being, bolsters executive functions, and augments the abilities for affective self-regulation, mitigating intense emotional states and refining interpersonal dynamics (95). Interventions based on MBI significantly elevated attention and emotional control, thereby improving MPA (96). Research has discerned that mental meditation interventions targeting MPA significantly tempered addictive behaviors, ameliorated self-regulation, and reduced stress (97). Among these methods, CBT underscores the transformation of cognition and behaviors, whereas mindfulness and meditation focus predominantly on accepting circumstances and emotions.

### 6.2 Exercise therapy

Youths engaged in social interaction and sports activities exhibited lower frequency and duration of mobile phone use (98), whereas individuals disinclined towards physical activity were more susceptible to developing MPA compared to their active counterparts (99). Research has demonstrated a decline in MPA levels among both aerobic exercise and Tai Chi groups (Tai Chi and Baduanjin are traditional Chinese aerobic exercises) (100). Impaired executive functioning was identified as a pivotal factor contributing to MPA (101), and moderate-intensity aerobic exercise has been shown to enhance executive functions in individuals with MPA (19), thereby facilitating addictive behaviors (102). Similarly, both basketball and Baduanjin exercises have been found to reduce levels of MPA (103).

### 6.3 Technological intervention

Transcranial direct current stimulation (tDCS) represents a non-invasive neuromodulation technique involving the placement of electrodes at two or more sites on the scalp, where a low-intensity direct current (1.0-2.0 mA) is administered to modulate cortical neuronal activity, effectively mitigating the psychological dependencies associated with addictive behaviors (104). DLPFC was pivotal in response inhibition (105), and stimulation of the DLPFC via tDCS could enhance an individual’s response inhibition capacity, thereby reducing dependency on mobile phones (20).

To furnish a more objective and continuous assessment and intervention, Lee (106) conceptualized a technological intervention dubbed the Smartphone Addiction Management System (SAMS). This system monitored users’ usage of mobile phone applications and transmitted the data to a backend server for analysis. Based on the results, specific feedback interventions were provided. Moreover, preliminary research by another team (13) has revealed that acupuncture therapy can restore the rhythmicity and quantity of representative salivary metabolites in patients with MPA, significantly facilitating sleep disturbances and alleviating symptoms of MPA, marking it as one of the effective therapeutic modalities. **Table 2** shows the interventions for MPA.

## 7 Conclusions

This review of the literature on MPA has explored a wide range of research examining its psychological abnormalities, dysfunctions in the neurobiological reward systems, weakened inhibitory control, cognitive impairments, genetic factors and disruptions in circadian rhythm. The impact of MPA on an individual’s psychophysiological well-being is significant, leading to a range of negative effects, including sleep disturbances and disrupted eating habits.

While a substantial body of research exists on MPA, several knowledge gaps remain. While there is ongoing debate regarding the specific pathology of MPA, including its relationship with anxiety, this review has defined both anxiety and depression as potential risk factors for MPA. Further investigation is needed into the long-term consequences of MPA, particularly its impact on brain development, cognitive function, and overall health outcomes. Additionally, the current research predominantly relies on cross-sectional studies utilizing self-reported data and the subjects are mostly Asians, which limits the ability to definitively establish causality between MPA and its associated factors. Moreover, the diagnostic criteria for MPA require global confirmation, encompassing diverse age groups, including young and elderly populations. We have concluded the review in the graph abstract.

In conclusion, the aim of this review was to provide insights into the emerging disease of Major Depressive Disorder (MPA), contributing to our understanding of its risk factors, pathophysiology, assessment, and treatment. We have presented a comprehensive overview of MPA. However, it is important to acknowledge the limitations that may influence the interpretation of these findings. To mitigate these limitations, we recommend that future research extend beyond examining the individual consequences of MPA and investigate its impact on families and society. This necessitates exploring therapeutic interventions that originate from family, societal, and cultural perspectives to implement more widespread and effective preventive measures. Additionally, recognizing the significant influence of genetic factors on the development of addictive behaviors is crucial. While studies have identified genetic polymorphisms and DNA methylation as potential biomarkers, the specific gene markers for MPA remain elusive. Future research should delve into neurobiological and molecular genetics to identify these markers, facilitating the screening of at-risk populations and the development of personalized preventative strategies. By addressing these limitations and focusing on these future directions, research on MPA can contribute to a deeper understanding of this growing public health concern and the development of effective interventions to mitigate its negative consequences.
